# New Glycosalen–Manganese(III) Complexes and RCA_120_ Hybrid Systems as Superoxide Dismutase/Catalase Mimetics

**DOI:** 10.3390/biomimetics8050447

**Published:** 2023-09-21

**Authors:** Valeria Lanza, Graziella Vecchio

**Affiliations:** 1 Istituto di Cristallografia, Consiglio Nazionale delle Ricerche, Via Gaifami 18, 95125 Catania, Italy; valeria.lanza@cnr.it; 2 Dipartimento di Scienze Chimiche, Università di Catania, Viale A. Doria 6, 95125 Catania, Italy

**Keywords:** carbohydrate, salen, manganese, SOD mimetics, EUK-108, EUK-8

## Abstract

Reactive oxygen species are implicated in several human diseases, including neurodegenerative disorders, cardiovascular dysfunction, inflammation, hereditary diseases, and ageing. Mn^III^–salen complexes are superoxide dismutase (SOD) and catalase (CAT) mimetics, which have shown beneficial effects in various models for oxidative stress. These properties make them well-suited as potential therapeutic agents for oxidative stress diseases. Here, we report the synthesis of the novel glycoconjugates of salen complex, EUK-108, with glucose and galactose. We found that the complexes showed a SOD-like activity higher than EUK-108, as well as peroxidase and catalase activities. We also investigated the conjugate activities in the presence of Ricinus communis agglutinin (RCA_120_) lectin. The hybrid protein–galactose–EUK-108 system showed an increased SOD-like activity similar to the native SOD1.

## 1. Introduction

Oxidative stress occurs from the imbalance between radical species and antioxidant defences. It has been associated with various adverse health conditions, such as chronic inflammation, diabetes, cancer, neurodegenerative diseases (Parkinson’s disease, Alzheimer’s disease, ALS), and ageing [1,2].

Oxidative injury in cells is controlled and preserved through enzymatic and nonenzymatic antioxidant systems [3]. The superoxide dismutase (SOD), catalase (CAT), and peroxidase enzymes form the front line of defence against oxidative stress products. The main nonenzymatic antioxidants are vitamins (C and E), β-carotene, uric acid, and GSH.

In light of their impact on health, there has been an interest in targeting reactive oxygen species (ROS) in the development of redox medicine [4]. Many antioxidants and redox drugs have been administrated as therapeutic strategies for a variety of diseases [5]. The therapeutic potential of SOD enzymes has also been explored, and bovine CuZn–SOD preparations (Orgotein^®^) were administrated in the late 1970s to treat inflammatory diseases. Orgotein is only used as an anti-inflammatory drug in non-human animals [6]. MnSOD has also been investigated [7]. The therapeutical administration of SOD enzymes has several limitations, including the immunogenicity in the case of the SOD enzyme from non-human sources, the low intracellular uptake, short half-lives, administration route, and costs [8].

Low molecular weight complexes of redox metals (Fe, Cu, Mn) mimicking natural SOD enzymes have been widely reported [7,9,10]. SOD mimetics (SODm) would be potential therapeutics and useful probes to elucidate the physiopathologic role of intracellular superoxide radicals [10,11]. SODm have all been shown to positively affect the inflammatory state in lung epithelial cells in models of chronic obstructive pulmonary disease [12]. Mn^II^ or Mn^III^ complexes have been successfully studied as catalytic drugs among a variety of metal complexes, due to the low Fenton-based toxicity of manganese [13,14,15]. The Mn^II^ complexes of cyclic polyamines [15] aneN5 (1,4,7,10,13-pentaaza cyclopentadecane)-type ligands have been studied as SODm, and Imisopasem Manganese (M40403) and Avasopasem Manganese (M40419 or GC-4419) are the best systems in this family for diseases related to ROS dyshomeostasis. The GC-4419 is in a Phase 3 clinical trial to reduce side effects from anticancer therapy [16,17]. In 2020 Galera Therapeutics Inc. announced the phase II clinical trial with GC4419 for critical illness due to COVID-19 [18].

The Mn^II^ complexes of polyamine (salan-type) ligands have also been investigated as SOD mimetics [19]. The salan ligands have also been functionalized with cell-penetrating peptides to improve their cell uptake and antioxidant activity [19]. Other families of SODm include the Mn^III^ complexes of porphyrin– and Mn^III^–salen (H_2_salen = N,N′ bis(salicylidene)ethylenediamine)-type complexes [20,21]. The Mn^III^ complexes of a variety of porphyrins have been widely studied [14,15,22,23].

Mn^III^–salen derivatives have been developing as combined SOD/CAT mimetics mainly by Eukarion Company [24,25,26]. Eukarion (EUK)-8 (or EUK-108 when the ligand is CH_3_COO^-^ instead of Cl^−^) and EUK-134 (or EUK-113 when the ligand is CH_3_COO^-^ instead of Cl^-^) are the prototype molecules of this family of complexes and have been tested in several pathological conditions [5,27,28]. Currently, EUK-134 is a component of numerous anti-ageing skincare products for its antioxidant properties. Various salen-type complexes have been studied recently in in vitro and in vivo animal models of diseases closely related to oxidative stress, such as neurodegenerative diseases and cancer [25,29,30,31,32].

**Figure 1:**
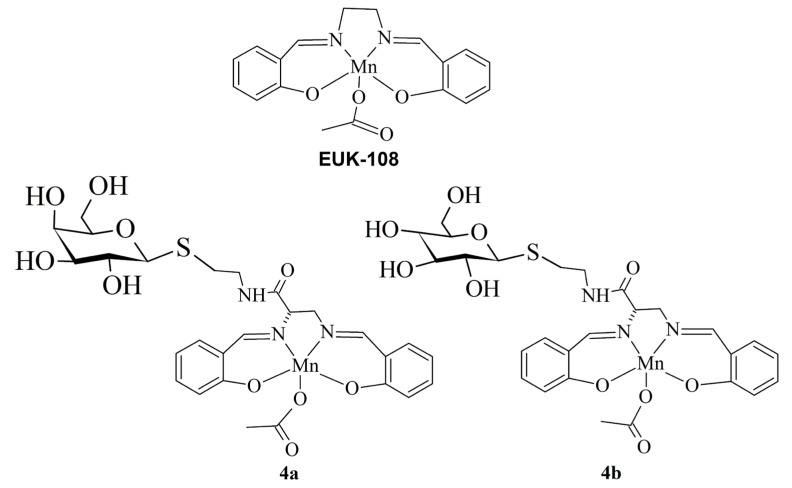
Structures of EUK-108 and Glycoconjugates synthesized in this study.

Based on the interest in salen derivatives [7,24,28,29,33,34], herein, we report the synthesis of two new Mn^III^ salen glycoconjugates. In particular, we conjugated an N,N′ bis(salicylidene) ethylenediamine derivative with glucose or galactose to synthesize EUK-108-analogous molecules (Figure 1).

We exploited the new features introduced by sugar moiety, such as the binding with specific lectins [35]. Ricinus communis agglutinin I (RCA_120_, RCA I) is a glycoprotein composed of two A and two B units linked by a disulfide bond. Subunit A is the catalytic unit and confers toxicity to the protein, while chain B binds carbohydrate chains with non-reducing terminal galactose residues [36]. RCA_120_ has low toxicity compared to the homolog ricin protein.

In the last few years, metal complexes have been caged in protein scaffolds to form artificial metalloenzymes, also called hybrids, and engineer new reactions [37,38,39,40]. Many anchoring strategies have been explored to build new artificial metalloenzymes. The properties of the scaffold protein can modulate the catalytic activity of the metal complex that acts as an artificial prosthetic group. The protein moiety provides a unique microenvironment (chirality, dielectric coefficient) and can improve solubility and substrate accessibility [37,38,39,40]. Some examples of the non-covalent strategy exploit the affinity of the metal complex for the protein, such as the streptavidine–biotin technology, which is applied in vivo for catalyzing abiotic reactions [41].

We synthesized non-covalent conjugates of RCA_120_ with galactose–salen conjugate **4a**. We investigated the SOD-like, peroxidase, and catalase activities of the metal complexes and the RCA_120_–glycosalen complexes.

The hybrid protein–galactose–salen system showed an increased SOD-like activity.

## 2. Materials and Methods

### 2.1. Material

Anhydrous DMF, anhydrous methanol, acetobromo-α-D-galactose, acetobromo-α-D-glucose, Nα, Nβ-Di-Boc-L-2,3-diaminopropionic acid (dicyclohexylamine salt) (DAPBoc), cysteamine (2-mercaptoethylamine, mea), o-(benzotriazole-1-yl)-N,N,N′,N′-bis(tetramethylene)O-(1H-benzotriazol-1-yl)-N,N,N′,N′-tetramethyluronium tetrafluoroborate (TBTU), 1-hydroxybenzotriazole (HOBT), and lectin from Ricinus communis (RCA_120_) were purchased from Sigma-Aldrich and were used without any purification. CM Sephadex C-25 (Sigma) (NH_4_^+^ form) and DEAE-Sephadex A-25 (HCO_3_^−^ form) were used for ion exchange chromatography. Ultra-pure water (Milli-Q Element, Millipore, Italy) was used for all the experiments. Thin layer chromatography (TLC) was carried out on silica gel plates (Polygram SIL G/UV254 0.2 mm Macherey-negel). Glycosidic derivatives were detected on TLC using UV or the anisaldehyde test.

EUK-108 was synthesized as reported elsewhere [42].

### 2.2. Synthesis of Salen Ligands and Mn^III^ Complexes

#### 2.2.1. Synthesis of 1-deoxy-1[(S-cysteamine)]-ß-galactose (**1a**) and of 1-deoxy-1[(S-cysteamine)]-ß-glucose (**1b**)

Mea (1.9 g, 24.6 mmol) and sodium methanolate (24.6 mmol) in water (2 mL) were added to a solution of α-D-acetobromo-galactose (2.42 g, 5.9 mmol) in DMF (2 mL). The reaction was carried out at 70 °C under stirring and nitrogen. After 3h, the solvent was evaporated. The product was obtained through the hydrolysis of acetyl groups in NaOH (1%) for 2h. The product was purified through reverse-phase RP8 column chromatography, and eluted using a NH_4_Cl solution (0.1 M). The appropriate fractions were collected and evaporated (Rf = 0.2, PrOH/H_2_O/AcOEt/NH_3_, 4:3:2:1). The solid residue was further purified by a CM Sephadex C-25 column (20 × 60 mm, NH_4_^+^ form) eluted using water and then a linear gradient of NH_4_HCO_3_ solution (400 mL) 0 → 0.3 M. Product **1a** was obtained, with a yield of 30%. Rf = 0.42 (PrOH/H_2_O/AcOEt/NH_3_ 4:3:2:1).

ESI-MS: *m*/*z* = 240.1 (**1a** + H)^+^

^1^H NMR (D_2_O, 500 MHz) δ(ppm): 4.37 (1H, d, J_H1,H2_ = 10.0 Hz, H-1 of Gal); 3.86 (1H, d, J_H3,H4_ = 4,0 Hz, H-4 of Gal); 3.67–3.56 (3H, m, H-5, H-6_a_, H-6_b_ of Gal); 3.54 (1H, dd, J_H3,H4_ = 4.0 Hz, J_H2,H3_ = 10.0 Hz, H-3 of Gal); 3.46 (1H, t, J_H1,H2_ = 10,0 Hz, H-2 of Gal); 2.80 (2H, m, CH_2_ in α to NH_2_); and 2.75 (m, 2H, CH_2_ in β to NH_2_).

The same procedure given for **1a** was followed to synthesize **1b** ), starting from α-D-acetobromo-glucose. The yield was 35%. Rf = 0.42 (PrOH/H_2_O/AcOEt/NH_3_ 4:3:2:1).

ESI-MS *m*/*z* = 240.0 (**1b** + H)^+^

1H NMR (D_2_O, 500 MHz) δ(ppm): 4.44 (1H, d, J_H1,H2_ = 10.0 Hz, H-1 of Glc); 3,80 (1H, dd, J_H5,H6_ = 9.0 Hz, J_H6,H6′_ = 12.0 Hz, H-6_a_ of Glc); 3,60 (1H, dd, J_H6a,H6b_ = 12.0 Hz, H-6_b_ of Glc); 3.38 (2H, m, H-3, H-5 of Glc); 3.31 (1H, t, J_H4,H5_ = 9.0 Hz, H-4 of Glc); 3.23(1H, t, J_H1,H2_ = 10.0 Hz, H-2 of Glc); 2,83 (2H, m, CH_2_ in α to NH_2_); and 2.73 (2H, m, CH_2_ in β to NH_2_).

#### 2.2.2. Synthesis of 1-deoxy-1[(S-cysteamidopropyl(1,2-diamino)]-ß-galactose] **2a** and 1-deoxy-1[(S-cysteamidopropyl(1,2-diamino)]-ß-glucose] **2b**

DAPBoc (250 mg, 0.82 mmol), TBTU (315mg, 0.82 mmol), and HOBT (111 mg, 0.82 mmol) in dry DMF (30 mL) were stirred under nitrogen at 25 °C. After 15 min, **1a** was added (200 mg, 0.82 mmol) to the solution. The reaction was stopped when the reagents disappeared in TLC (PrOH/H_2_O/AcOEt/NH_3_, 5:2:1:1). DMF was evaporated and the solid was purified through a reverse-phase C-8 column with a gradient of H_2_O/acetone (0→50%). The Boc group was removed with CF_3_COOH for 1h. CF_3_COOH was evaporated and the product was passed through a DEAE-Sephadex A-25, using water as the eluent. Rf = 0.31 (PrOH/H_2_O/AcOEt/NH_3_,5:2:2:2); and the yield was 40%.

ESI-MS *m*/*z* = 326.4 (**2a** +H)^+^

^1^H NMR (D_2_O, 500 MHz) δ(ppm): 4.40 (1H, d, J_H1,H2_ = 10.0 Hz H-1 of Gal); 3.88 (1H, d, J_H3-H4_ = 3.0 Hz, H-4 of Gal); 3.70–3.58 (3H, m, H-5, H-6_a_, H-6_b_ of Gal); 3.55 (1H, dd, J_H2-H3_ = 10.0 Hz, J_H3,H4_ = 3.0 Hz, H-3 of Gal); 3.46 (1H, t, J_H1,H2_ = 10.0 Hz, H-2 of Gal); 3.40 (3H, m, CH_2_ in b to S, CH of DAP); 2.85 (1H, m, CH_2_ of DAP); and 2.78 (4H, m,CH_2_ in a to S and CH_2_ of DAP).

**2b** was synthesized as reported for **2a**

ESI-MS m/z = 326.6 (**2b** + H)^+^

^1^H NMR (D_2_O, 500 MHz) δ (ppm): 4.36 (1H, d, J_H1,H2_ = 10.0 Hz H-1 of Glu); 3.84 (1H, d, J_H3,H4_ = 10.0 Hz H-4 of Glc); 3.68–3.56 (3H, m, H-5, H-6_a_, H-6_b_ of Glc); 3.53 (1H, dd, J_H2,H3_, = 4.0 Hz, J_H3-H4_, = 10.0 Hz, H-3 of Glc); 3.46 (H, t, J_H,-H 2_= 10.0 Hz, H-2 of Glc); 3.38 (3H, m, CH_2_ in β to S, CH of DAP); 2.82 (2H, m, CH_2_ of DAP); and 2.75 (4H, m, CH_2_ in α to S).

#### 2.2.3. Synthesis of 1-deoxy-1[(S-cysteamidopropyl(1,2-diamino)N,N′-bis(salicylidene))]-ß-galactose] **3a**

**2a** (50 mg, 1.5 mmol) was dissolved in anhydrous methanol (5 mL) and a stoichiometric amount of salicylaldehyde (12.5 mL, 3.0 mmol) was added under stirring at 25 °C. A yellow solid was formed. After 6 h, the solvent was evaporated under vacuum and the solid obtained was washed with hexane/acetone (1:1). The yield was 88%.

ESI-MS *m*/*z* = 534.5 (**3a** + H)^+^

^1^H NMR (CD_3_OD, 500 MHz) δ (ppm): 8.54 (s; 1H, m, imine H); 8.45(s; 1H, s, imine H); 7.32 (4H, m, H-6 and H-3); 6.82–6.83 (4H, m, H-4 and H-5); 4.33 (1H, d, J_XY_ = 6.0 Hz CH in α to O=C); 4.31 (1H, d, J_1,2_ = 9.70 Hz Gal H-1and CH); 4.15 (2H, m, CHa in β to O=C-NH); 4.05 (2H, m, CHb in β to O=C-NH); 3.84 (1H, d, J_H3,H4_ = 5.0 Hz, Gal H-4); 3.75 (1H, m, Gal H-5); 3.46 (1H, dd, J_H2,H3_ = 11.0 J_H3,H4_ = 5.0 Hz, Gal H-3); 3.57–3.48 (3H, m, Gal H-6, H-6′, H-2); 3.42 (2H, m, CH_2_ in α to-NH-C=O); 2.89 (2H, m, CH_a_ in β to -NH-C=O); and 2.77 (2H, m, CHb in β to O=C-NH). The glucose–salen conjugate **3b** was synthesized as reported for **3a**.

ESI-MS *m*/*z* = 534.0 (**3b** + H)^+^

^1^H NMR (CD_3_OD, 500 MHz) δ(ppm): 8.53 (1H, s, imine H); 8.45 (1H, s, imine H); 7.43–7.20 (4H, m, H-6 and H-3); 6.97–6.82 (4H, m, H-4 and H-5); 4.36 (1H, d, J_H1,H2_ = 9.7 Hz, Glc H-1); 4.33 (1H, m, CH in α O=C-NH); 4,14 (2H, m, CHa in β to O=C-NH); 4.05 (2H, m, CHb in β to O=C-NH); 3.84 (1H, d, J_H3,H4_ = 5.0 Hz, Glc H-4); 3.84 (1H, d, J_3,4_ = 12.0 Hz, Glc H-6); 3.61–3.45 (5H, m, Glc H-5, H-4, H-6′, CH_2_ in α to -NH-C=O); 3.26 (1H, m, Glc H-3); 3,19 (1H, t, J_H1-H2_ = 10,0 Hz, Glc H-2); 2.88 (2H, m, CHa in β to -NH-C=O); and 2.72 (2H, m, 2H, CHb in β to O=C-NH).

#### 2.2.4. Synthesis of Manganese(III) Complexes of **3a** and **3b**

**3a** (115 mg, 0.086 mmol) and NaOH (0.172 mmol, methanolic solution) were dissolved in anhydrous methanol (5 mL). Mn(CH_3_COO)_2_ (0.086 mmol) was added to the solution under stirring. The yellow solution turned brown and the reaction mixture was refluxed. After 3 h, the precipitate was filtered off and washed with acetone. The solid was further purified through precipitation from a water and acetone solution.

The yield was 90%.

**4a:** ESI-MS m/z = 586.2 [**4a**-Ac]^+^

**4b** was synthesized as reported for **4a**.

**4b:** ESI-MS m/z = 586.0 [**4b**-Ac]^+^

#### 2.2.5. Preparation of Hybrid Mn^III^ and RCA_120_ System

**4a**- or **4b**-RCA_120_ adducts were prepared by incubating equimolar amounts of RCA_120_ lectin and Mn complex for 10 min in the buffer solution, at 25 °C.

### 2.3. Instrumentation

The NMR spectra were recorded at 25 °C with a Varian Inova 500 spectrometer. The ^1^H NMR spectra were acquired using standard Varian library pulse programs. The 2D spectra (COSY, TOCSY, HSQC) were acquired using 1K data points, 256 increments, and a relaxation delay of 1.5 s.

The mass spectra were recorded with a Mariner Perseptive Biosystem ESI-MS.

The UV-visible spectra were recorded with an Agilent 8452A diode array spectrophotometer. The CD spectra were performed on a JASCO model J-810 spectropolarimeter.

The **4a**, **4b,** and EUK-108 stock solutions were prepared in EtOH/water (1:1). The final EtOH concentration did not exceed 5% and did not affect the measurements. The spectra of freshly prepared solutions were recorded at 25 °C.

### 2.4. Superoxide Dismutase Assay

The SOD-like activity was determined using the indirect method [43]. Superoxide anion was generated by the xanthine/xanthine oxidase system and spectrophotometrically detected by monitoring the nitro blue tetrazolium (NBT) reduction to formazan at 560 nm, for 600 s.

The amount of xanthine oxidase required to produce a ΔA_560_ nm/min = 0.024 was added to 2 mL of the reaction mixture (NBT 250 μM, xanthine 50 μM, phosphate buffer 0.010 M, pH 7.4), as reported elsewhere [44]. The ΔA_560_ = 0.024 nm/min corresponds to the 1.1 mM/min^·^ O_2_ production rate. The NBT reduction rate was also measured in the presence of the Mn^III^ complexes (concentration ranging from 10^−5^ to 10^−8^ M). All measurements were carried out at 25 °C under stirring. In separate experiments, urate production was spectrophotometrically monitored at 295 nm to exclude the inhibition of xanthine oxidase activity in the presence of Mn^III^ complexes or RCA_120_.

The I_50_ value (the concentration of metal complex required to inhibit 50% of NBT reduction) was determined.

### 2.5. Catalase Activity Assay

The catalase activity was determined as reported elsewhere [34]. An H_2_O_2_ solution (30 mM) was incubated at 25 °C with theMn^III^ complex (concentration ranging from 10^−5^ to 10^−6^ M) in 0.010 M phosphate buffer (pH 7.4).

ABTS (2,2-azino-bis(3-ethylbenzothiazoline-6-sulfonic acid) (50 mM) and horseradish peroxidase (1.3 mg/mL) were added after 50 min. The absorbance at 735 nm was measured after 5 min, and the amount of H_2_O_2_ consumed per min per mmole from the compound was determined.

### 2.6. Peroxidase Activity Assay

The peroxidase activity of **4a**, **4b,** and EUK-108 compounds was assayed by monitoring spectrophotometrically the H_2_O_2_-dependent oxidation of ABTS [45]. The ABTS (0.2 mM), Mn^III^ complex (10 mM), and H_2_O_2_ (0.5 mM) in phosphate buffer (50 mM, pH 7.4) were incubated at 25 °C.

ABTS oxidation was monitored at 735 nm (ε_ABTS_^+^ = 15,000) where the metal complexes did not show any absorption. No oxidation reaction of ABTS with the complexes was observed in the assay condition.

## 3. Results and Discussion

### 3.1. The Ligands

**Figure 2:**
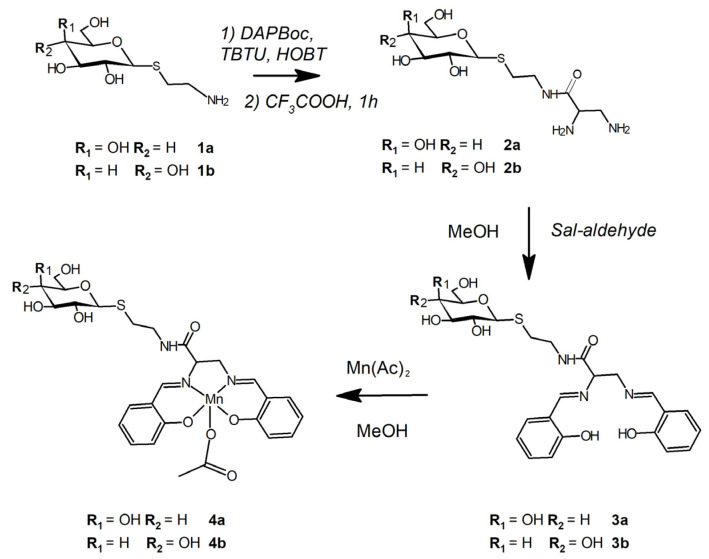
Synthesis scheme of **4a** and **4b**.

**4a** and **4b** were synthesized from sugar amino-derivatives via the multistep procedure reported in Figure 2. The anomerically pure **1a** and **1b** were obtained in good yield through a nucleophilic substitution reaction between α-D-acetobromo-glycoside and cysteamine in water at basic pH. The amino group of **1a** and **1b** was linked to DAPBoc through a condensation reaction, and the products were purified using reverse-phase chromatography.

After removing the Boc-protecting groups through acid hydrolysis, the derivatives **2a** and **2b** were obtained and isolated through ionic-exchange chromatography. **3a** and **3b** were synthetized from **2a** and **2b** and sal-aldehyde at 25 °C in anhydrous methanol.

The NMR and ESI-MS spectra confirmed the identity of the products and intermediates (Figure S1–S13). In the ^1^H NMR spectrum of **1a** and **1b,** the signals of the sugar unit and the ethylenic chain were present. The chemical shift of the H-1 signal and the coupling constant values (10.0 Hz) confirmed the β-configuration of the anomeric carbon of the sugar residue. In the ^1^H NMR spectra of **2a**, the signals of the galactose protons were assigned using the COSY and TOCSY spectra. The protons of the cysteamine moiety resonated at 3.50 ppm and at 2.78 ppm, while the protons of the ABX system of the propionyl chain resonated at 3.40 ppm (X), 2.83 and 2.80 ppm (A, B). A similar trend was found in the **2b** spectrum.

In the ^1^H NMR spectra of **3a**, the imino protons appeared at 8.60 and 8.49 ppm. The signal at 8.60 ppm was assigned to the imino group α to the amido group. The chemical shifts of the protons of the two benzene rings also resonated differently. The signals of the ABX system of the diamino–propionyl chain were evident in the spectra. The spectra of **3b** were similar to the spectra of **3a.**

### 3.2. The Manganese(III) Complexes

The Mn^III^ complexes **4a** and **4b** were synthesized, as reported for salen complexes [46], at 25 °C and were isolated by adding acetone to the reaction mixture. The UV–Vis spectra of **4a** and **4b** were not significantly different from that of the parent salen complex EUK-108. In the spectra, there were the typical transitions at 323 nm, due to π → π* C=N groups and at 388 nm, due to LMCT, suggesting the same coordination environment of the Mn^III^ compared to EUK-108. In the ESI mass spectra (Figure S13), a leading peak corresponded to the complex species [Mn^III^ complex]^+^ (**4a** or **4b** without CH_3_COO^−^ ligand).

### 3.3. SOD-like Activity

**Figure 3:**
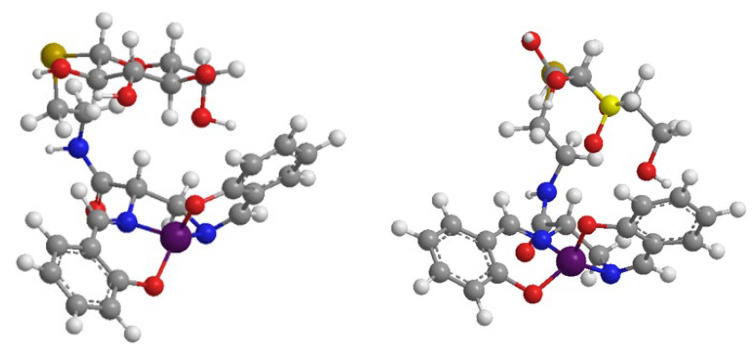
Molecular modelling of **4a** (**left**) and **4b** (**right**).

The reaction of **4a** and **4b** with the superoxide anion was determined by competition kinetic experiments with NBT being used as the target molecule [47,48]. Plotting Vo/Vcat-1 versus the complex concentration produced a straight line with a slope k_cat_/k_NBT_x[NBT] [49] (Figure S14), where Vo was the NBT reduction rate Vcat was the NBT reduction rate in the presence of the investigated complex, and k_NBT_ was 5.88 × 10^4^ M^−1^s^−1^ [43,50]. The I_50_ and k_cat_ values are reported in Table I. **4a** and **4b** showed good SOD-like activity, and their I_50_ values were about ten times lower than that of EUK-108. The I_50_ values were similar to those reported for the EUK-108–cyclodextrin conjugate [44]. The effect of the modification of the salen–diamine bridge on catalytic activity has been explored in a few cases [26,45,51]. In the case of the cyclodextrin derivatives previously studied, we hypothesized the role of the hydrophobic cavity and/or OH groups in order to explain the improved SOD-like activity. The results on monosaccharide conjugates may suggest the prominent role of OH groups in improving the SOD-like activity of the catalytic center. Molecular modelling suggests that, in **4a** and **4b**, the sugar side-chain may bend toward the catalytic center (Figure 3), which could improve the SOD-like activity.

**4a** and **4b** activities were also investigated in the presence of RCA_120_. The **4a**/ or **4b**/RCA_120_ adducts were synthesized by pre-incubating equimolar amounts of RCA_120_ lectin and **4a** or **4b** for 15 min in the buffer solution at 25 °C.

The RCA_120_ alone did not show any interference with the SOD assay. The I_50_ value of the **4a**–RCA_120_ adduct was 5.0 × 10^−8^ M and the k_cat_ was 2.8 × 10^8^ M^−1^ s^−1^ (Table 1, Figure S15). These values were significantly higher than those of the free **4b** and they were of the same order of magnitude as that of the native SOD1 enzyme (1.4 × 10^8^ M) [52]. No activity improvement was observed when **4b** was pre-incubated with RCA120 [8].

We hypothesized that RCA_120_ could bind **4a,** as reported for other galactose conjugates [35,53], and that the protein environment could modulate the activity of the catalytic center, as found for other SOD-mimetic hybrid systems [39,54,55,56]. The interaction between the RCA_120_ and **4a** complex was investigated using CD spectroscopy. In the UV region of the CD spectra (Figure S16), RCA_120_ showed a positive peak at 190 nm and negative peaks at 208 nm and 220 nm, in keeping with the partial *α*-helix structure of RCA_120_ (15%) [53]. The intensity of the signals was slightly modified after the addition of the **4a** complex to RCA_120_ (Figure S16), suggesting the interaction of **4a** with the protein. No modification of the CD spectrum of RCA_120_ was found when **4b** was added to RCA_120_ (Figure S17). These data can explain the effect on the SOD activity in the presence of the RCA_120_ protein, which can only bind the **4a** complex.

### 3.4. Catalase Activity

Both salen complexes **4a** and **4b** exhibited a similar catalase activity, and their activities (Table 2) were slightly lower than EUK-108’s [45].

The reaction mechanism by which the Mn complexes act as catalase mimetics has been investigated [45]. It involves the oxidation of Mn^III^ by H_2_O_2_ to oxo–manganese and the formation of H_2_O. The oxo–manganese complex is then reduced to Mn^III^ by another H_2_O_2_ molecule to form O_2_.

The presence of RCA_120_ slightly changed the CAT activity of both complexes.

### 3.5. Peroxidase Activity

The peroxidase activities of **4a**, **4b,** and EUK-108 were determined using the ABTS assay [29,30,34]. ABTS was the model substrate which reacted with H_2_O_2_

2 ABTS + H_2_O_2_ + 2H+ = 2 ABTS·+ + H_2_O

Mn complexes can catalyze the oxidation of ABTS with H_2_O_2_ (peroxidase activity). The mechanism for salen–Mn^III^ complexes consisted in the formation of the oxo–manganese complex intermediate, which could oxidate the ABTS generating ABTS^+^·[57,58,59,60].

The UV-vis spectrum of the green ABTS^·+^ radical cation showed characteristic bands at 415, 650, 735, and 815 nm. The solution of ABTS and H_2_O_2_ without the Mn complex was stable for several hours at 25 °C [33].

The amount of the ABTS^+^ formed per minute (µM ABTS/min) at the concentration of the complex 1.0 × 10^−5^ M is reported in Table 2.

The two Mn^III^ glycosalen complexes showed similar peroxidase activity compared to EUK-108.

The RCA_120_–**4a** adduct slightly improved the peroxidase activity compared to **4a** (Table 2). No effect of RCA120 was found for **4b**.

## 4. Conclusions

Antioxidant enzymes play a crucial role in physiological and pathological states. SOD mimetics may provide therapeutic strategies in redox medicine to reduce the severity of diseases related to oxidative stress. A family of SOD/Catalase mimetics are Mn^III^ complexes of salen-type ligands.

We synthesized new glycoconjugates of EUK-108 and their Mn^III^ complexes. The sugar moiety increased the solubility in the water of the system compared to that of the free EUK-108. The glycoconjugation improved the efficiency of SOD mimetics by about ten times compared to that of the free EUK-108. Instead, the glycoconjugates showed a similar catalase and peroxidase activity to the EUK-108. The sugar moiety conferred new properties to the conjugates, such as the binding with specific lectins. We found that the galactose derivative interacted with RCA_120_ lectin, which can selectively recognize galactose units. The hybrid system galactose–EUK-108–RCA_120_ lectin showed five times better SOD-like activity compared to that of the free galactose–EUK-108 conjugate. The hybrid system showed a SOD activity similar to that of the native SOD1 enzyme. The RCA_120_ protein environment could modulate the activity of the catalytic center.

These results disclose the potential of supramolecular mimetics. Based on the interest in antioxidant enzyme mimetics to counteract oxidative stress, this approach could be helpful for further improvement of the SOD mimetics.

## Figures and Tables

**Table 1:** SOD-like activity in the Fridovich assay (NBT = 250 μM) of **4a**, **4b,** and their RCA_120_ adducts.

Complex	I_50_ (μM)	k_cat_ (M^−1^ s^−1^)
**EUK-108**	2.05 (±0.03)	(7.2 ± 0.9) × 10^6^
**4a**	0.20 (±0.04)	(7.4 ± 0.7) × 10^7^
**4b**	0.25 (±0.05)	(5.9 ± 0.4) × 10^7^
**4a/RCA_120_**	0.05 (±0.01)	(2.9 ± 0.3) × 10^8^
**4b/RCA_120_**	0.20 (±0.03)	(8.0 ± 0.4) × 10^7^

**Table 2:** Catalase and peroxidase activity of **4a**, **4b,** and their RCA_120_ adducts.

Complex	CAT Activity	Peroxidase Activity
	µmol H_2_O_2_^/^min/mmol	µM ABTS/min
**EUK108**	32.2 (±1.7)	18.3 (±0.7)
**4a**	40.8 (±1.5)	21.6 (±0.6)
**4b**	40.6 (±1.2)	23.3 (±0.5)
**4a/RCA_120_**	31.5 (±1.5)	35.7 (±0.7)
**4b/RCA_120_**	28.8 (±1.2)	25.3 (±1.0)

## Data Availability

The authors confirm that the data supporting the findings of this study are available within the article and its supplementary materials.

## Supplementary Materials

The following supporting information can be downloaded at: https://www.mdpi.com/article/10.3390/biomimetics8050447/s1, Figures S1–S13: NMR and ESI spectra; Figures S14 and S15: SOD-like activity; Figures S16-S17: CD spectra of RCA_120_.
