# Supplementary material for: New Glycosalen–Manganese(III) Complexes and RCA120 Hybrid Systems as Superoxide Dismutase/Catalase Mimetics
Source: Biomimetics (Basel). 2023 Sep 21;8(5):447. doi: 10.3390/biomimetics8050447 (PMC10527547; doi:10.3390/biomimetics8050447)
Supplement: Supplementary file 1 [file biomimetics-08-00447-s001.zip › biomimetics-2525578-supplementary.pdf]

New Glycosalen– Manganese(III) Complexes and RCA_{120} Hybrid Systems as Superoxide Dismutase/Catalase Mimetics

[1] Istituto di Cristallografia, Consiglio Nazionale delle Ricerche, Via Gaifami 18, 95125 Catania, Italy; valeria.lanza@cnr.it
[2] Dipartimento di Scienze Chimiche, Università di Catania, Viale A. Doria 6, 95125 Catania, Italy

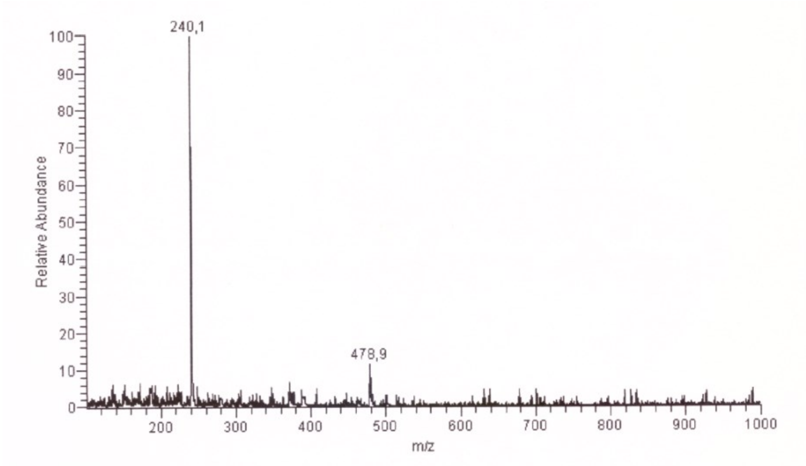

Figure S1. ESI-MS spectrum of **1a**

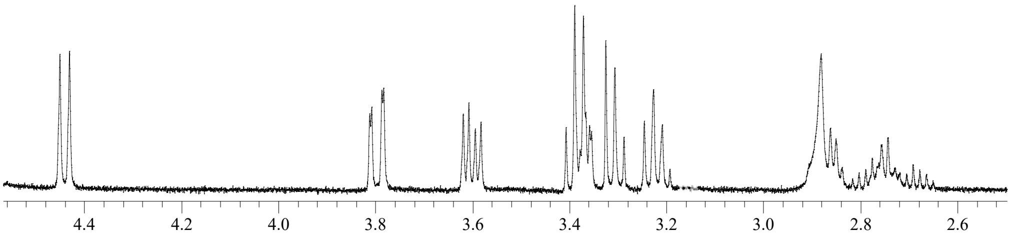

Figure S2. ^{1}H NMR spectrum of **1a** (D_2O, 500 MHz)

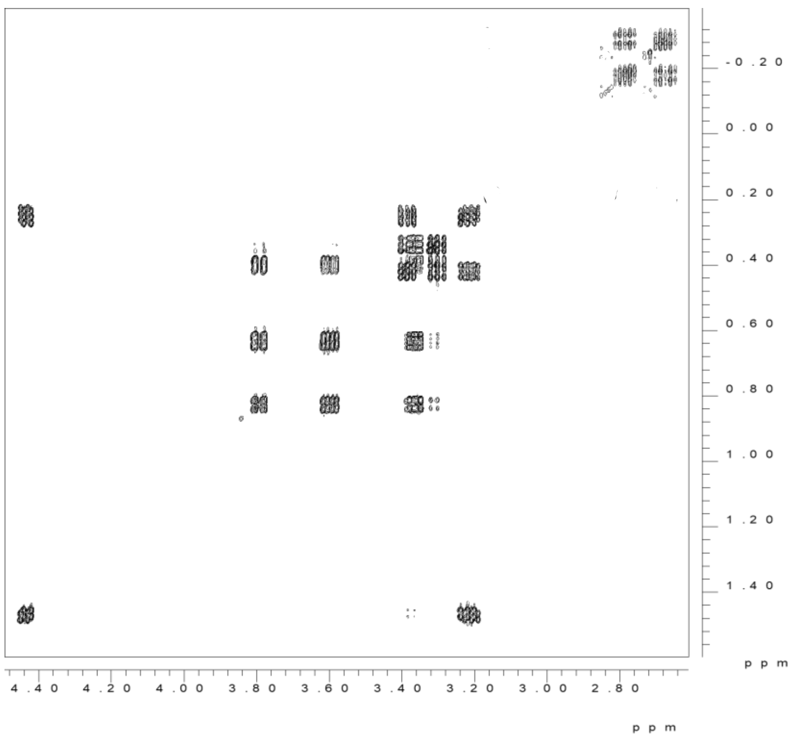

Figure S3. ^{1}H NMR COSY spectrum of **1a** (D_2O, 500 MHz)

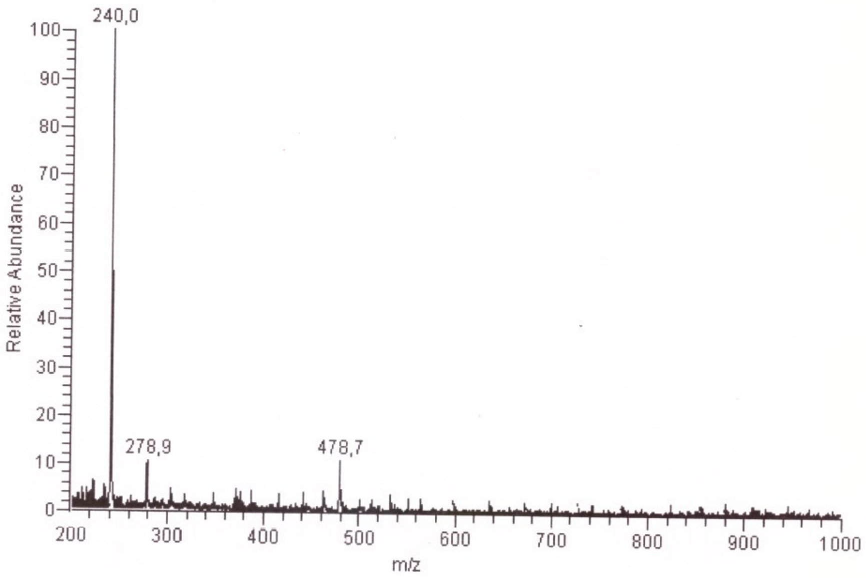

Figure S4. ESI-MS of **1b**

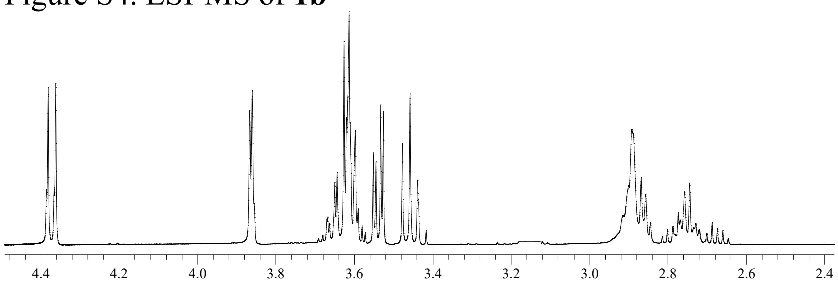

Figure S5. ^{1}H NMR spectrum of **1b** (D_2O, 500 MHz)

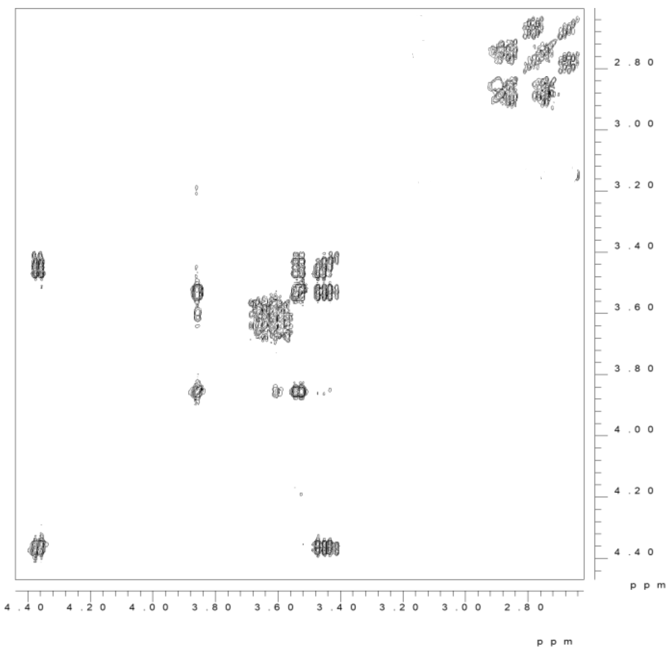

Figure S6. ^{1}H NMR COSY spectrum of **1b** (D_2O, 500 MHz)

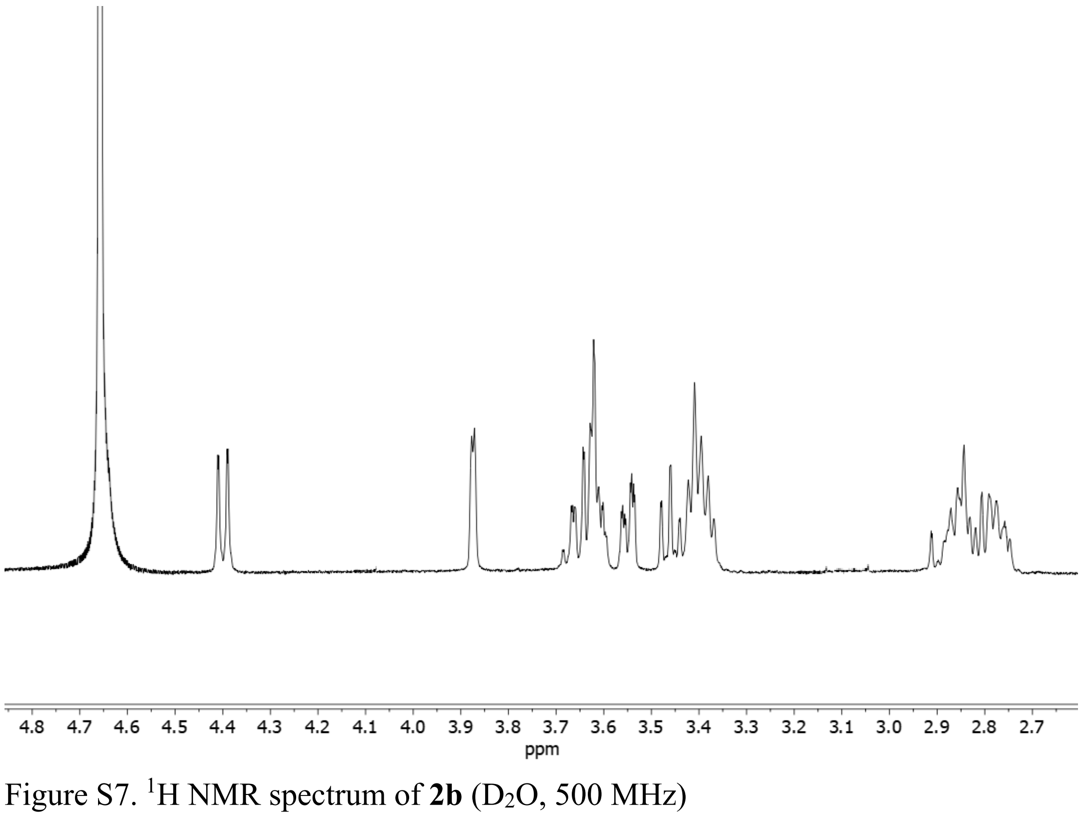

Figure S7. ^{1}H NMR spectrum of **2b** (D_2O, 500 MHz)

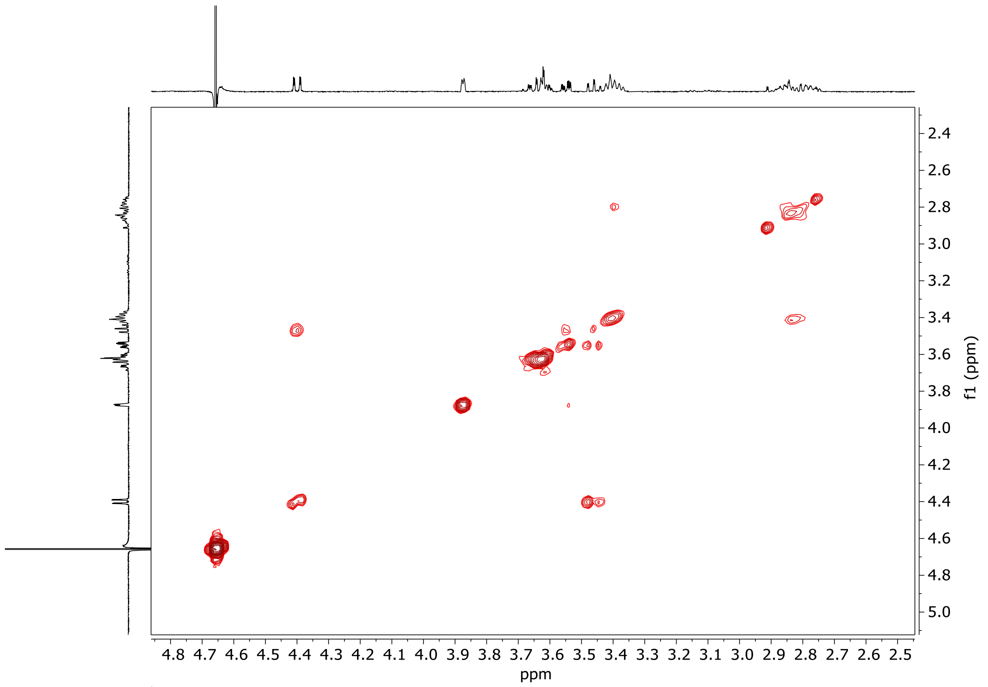

Figure S8. ^{1}H NMR COSY spectrum of **2b** (D2O, 500 MHz)

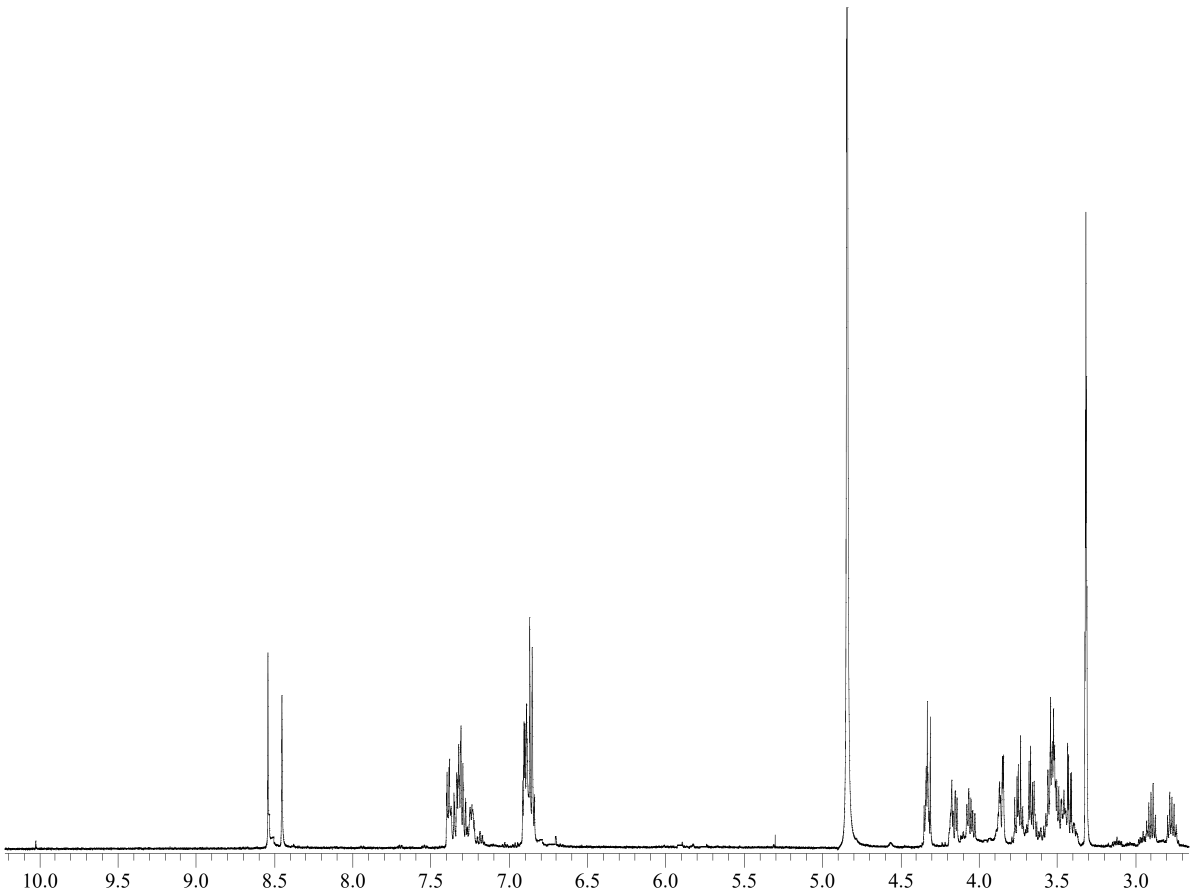

Figure S9. ^{1}H NMR spectrum of **3a** (CD_3OD, 500 MHz)

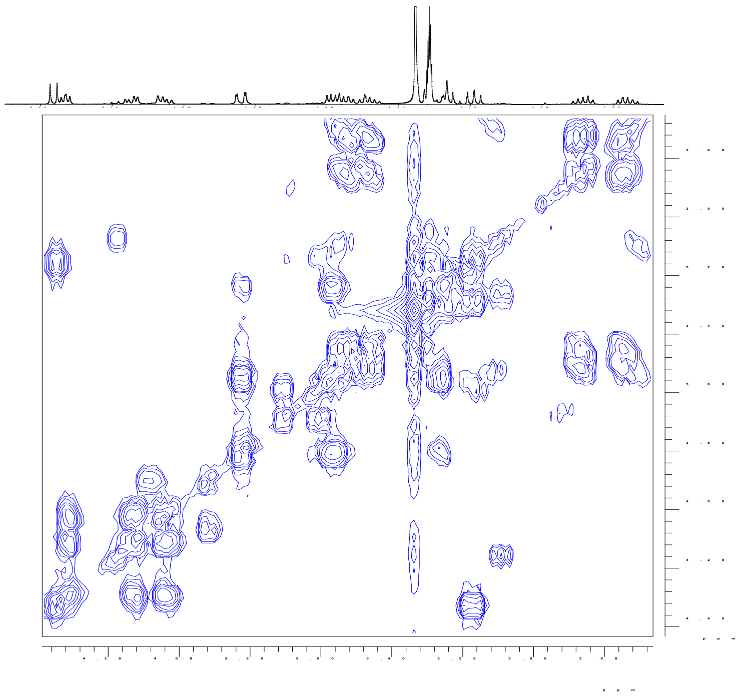

Figure S10. ^{1}H NMR COSY spectrum of **3a** (CD_3OD, 500 MHz)

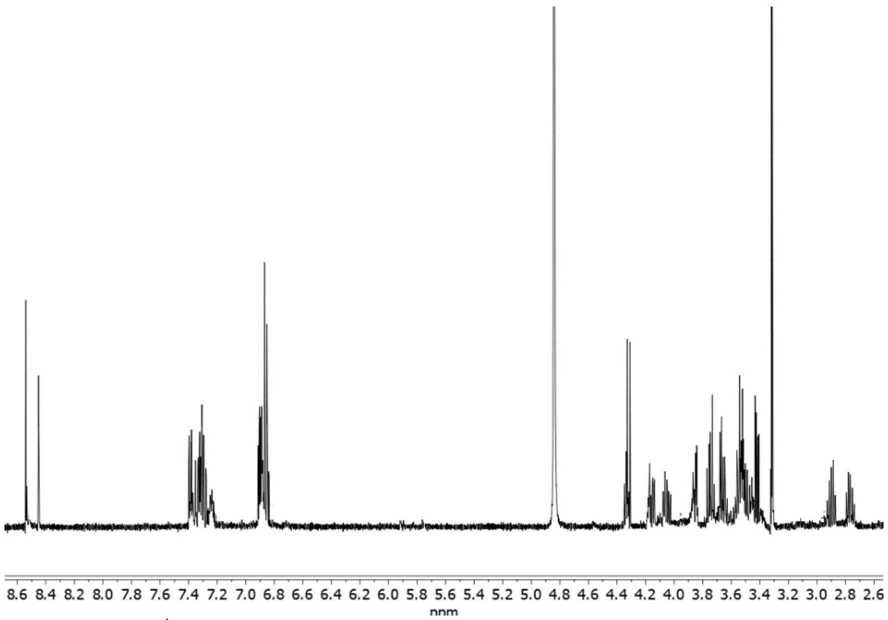

Figure S11. ^{1}H NMR spectrum of **3b** (CD_3OD, 500 MHz)

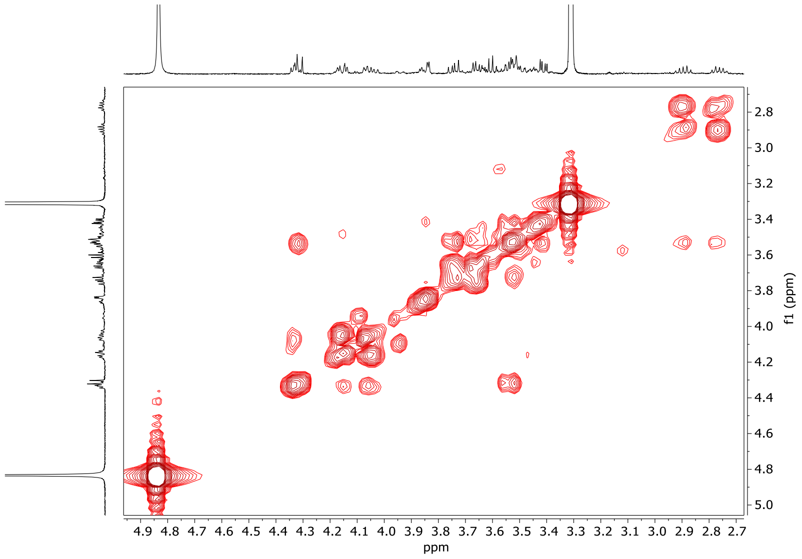

Figure S12. ^{1}H NMR COSY spectrum of **3b** (CD_3OD, 500 MHz)

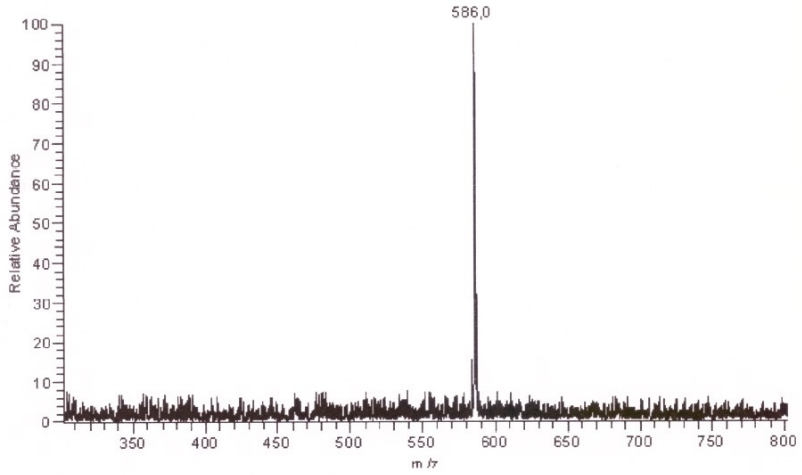

Figure S13. ESI-MS of **4b**

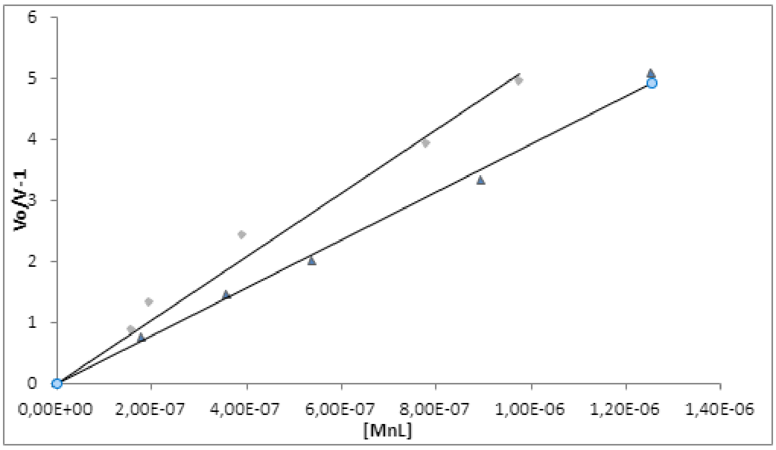

Figure S14. SOD like activity of **4a** (diamond) and **4b** (triangle) (Fridovich assay).

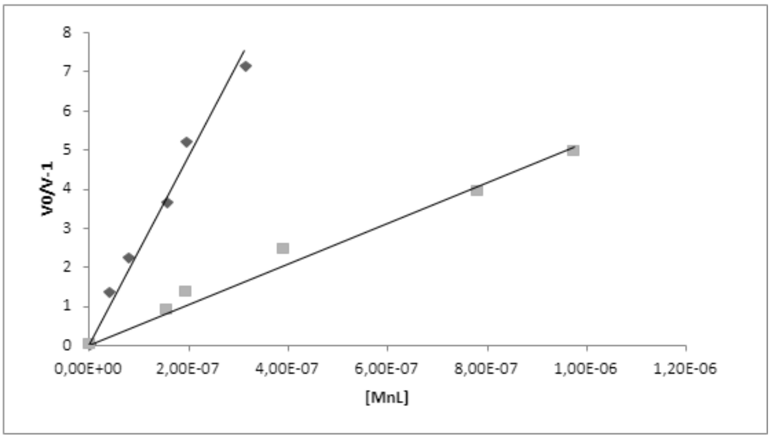

Figure S15. Comparison between SOD-like activity of **4a** alone (gray squares) and after incubation with RCA_{120} (black squares).

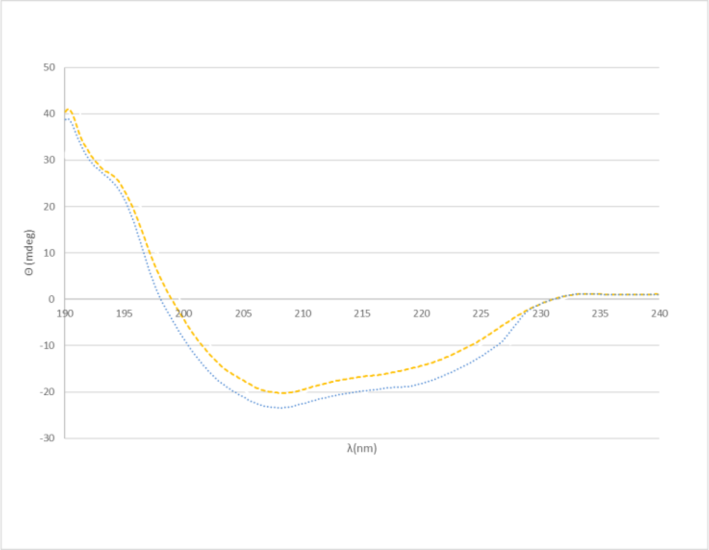

Figure S16. CD spectra of RCA_{120} alone ($1.6x10^{-4}$ M) (blue) and in the presence of **4a** ($3.2x10^{-4}$ M) (yellow).

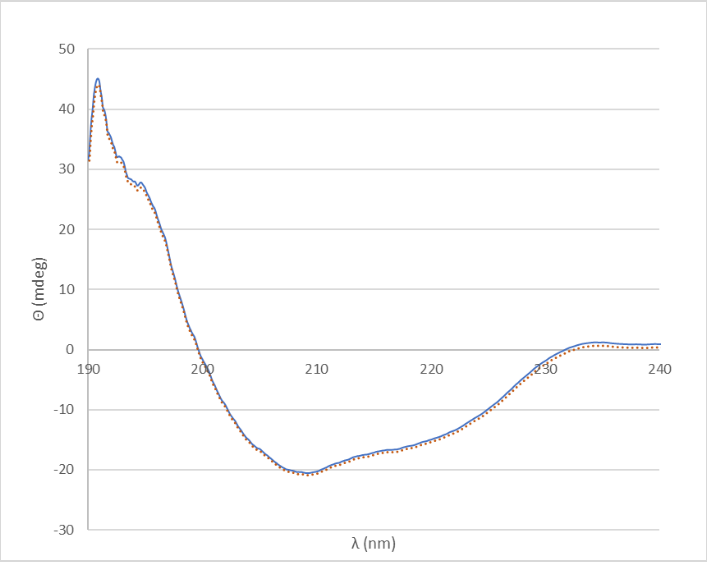

Figure S17. CD spectra of RCA_{120} alone ($1.6x10^{-4}$ M) (blue) and in the presence of **4b** ($3.2x10^{-4}$ M) (red).
